# 1-{4-Chloro-2-[2-(2-fluoro­phen­yl)-1,3-dithio­lan-2-yl]phen­yl}-2-methyl-1*H*-imidazole-5-carbaldehyde

**DOI:** 10.1107/S1600536811002595

**Published:** 2011-01-26

**Authors:** Hoong-Kun Fun, Suchada Chantrapromma, V. Sumangala, G. K. Nagaraja, Boja Poojary

**Affiliations:** a X-ray Crystallography Unit, School of Physics, Universiti Sains Malaysia, 11800 USM, Penang, Malaysia; b Crystal Materials Research Unit, Department of Chemistry, Faculty of Science, Prince of Songkla University, Hat-Yai, Songkhla 90112, Thailand; c Department of Chemistry, Mangalore University, Mangalagangotri 574 199, Karnatak State, India

## Abstract

There are two mol­ecules in the asymmetric unit of the title imidazole derivative, C_20_H_16_ClFN_2_OS_2_. In one mol­ecule, the dithiol­ane ring is disordered over two positions in a 0.849 (9):0.151 (10) ratio. The imidazole ring makes dihedral angles of 79.56 (9) and 18.45 (9)° with the 4-chloro­phenyl and 2-fluoro­phenyl rings, respectively, in one mol­ecule; in the other mol­ecule, the corresponding angles are 82.72 (9) and 17.39 (10)°. In the crystal, mol­ecules are linked by weak C—H⋯O inter­actions and these linked mol­ecules are stacked along the *b* axis by π–π inter­actions with a centroid–centroid distance of 3.4922 (11) Å. In addition, π–π inter­actions between the imidazole and 2-fluoro­phenyl rings are also observed, with centroid–centroid distances of 3.4867 (11) and 3.4326 (10) Å. The crystal is further consolidated by weak C—H⋯π inter­actions. Cl⋯S [3.5185 (8) Å], C⋯O [3.192 (3) Å] and C⋯C [3.326 (2)–3.393 (3) Å] short contacts are also observed.

## Related literature

For reference bond-length data, see: Allen *et al.* (1987 ▶). For details of ring conformations, see: Cremer & Pople (1975 ▶). For background to and applications of imidazole derivatives, see: Dutta *et al.* (2009 ▶); Hori *et al.* (2000 ▶); Khabnadideh *et al.* (2003 ▶); Mamolo *et al.* (2004 ▶); Quattara *et al.* (1987 ▶); Sengupta & Bhattacharya (1983 ▶); Ucucu *et al.* (2001 ▶); Yesilada *et al.* (2004 ▶).
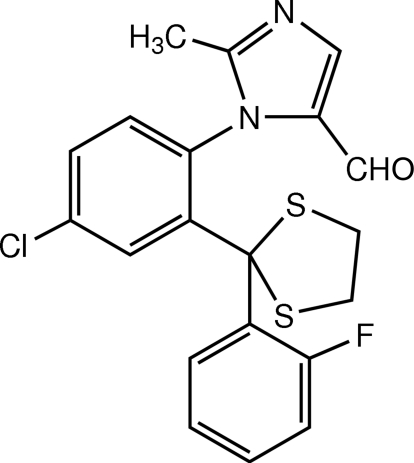


## Experimental

### 

#### Crystal data

C_20_H_16_ClFN_2_OS_2_
*M* *_r_* = 418.92
Monoclinic, 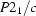

*a* = 18.5654 (3) Å
*b* = 9.2730 (1) Å
*c* = 24.7174 (4) Å
β = 116.807 (1)°
*V* = 3797.96 (10) Å^3^
*Z* = 8
Mo *K*α radiation
μ = 0.44 mm^−1^
*T* = 297 K
0.57 × 0.52 × 0.43 mm

#### Data collection

Bruker SMART APEXII CCD area-detector diffractometer
Absorption correction: multi-scan (*SADABS*; Bruker, 2005 ▶) *T*_min_ = 0.787, *T*_max_ = 0.833
42050 measured reflections
11058 independent reflections
8238 reflections with *I* > 2σ(*I*)
*R*_int_ = 0.025

#### Refinement

*R*[*F* ^2^ > 2σ(*F* ^2^)] = 0.040
*wR*(*F* ^2^) = 0.110
*S* = 1.01
11058 reflections
498 parameters
H-atom parameters constrained
Δρ_max_ = 0.47 e Å^−3^
Δρ_min_ = −0.29 e Å^−3^

### 

Data collection: *APEX2* (Bruker, 2005 ▶); cell refinement: *SAINT* (Bruker, 2005 ▶); data reduction: *SAINT*; program(s) used to solve structure: *SHELXTL* (Sheldrick, 2008 ▶); program(s) used to refine structure: *SHELXTL*; molecular graphics: *SHELXTL*; software used to prepare material for publication: *SHELXTL* and *PLATON* (Spek, 2009 ▶).

## Supplementary Material

Crystal structure: contains datablocks global, I. DOI: 10.1107/S1600536811002595/wn2418sup1.cif

Structure factors: contains datablocks I. DOI: 10.1107/S1600536811002595/wn2418Isup2.hkl

Additional supplementary materials:  crystallographic information; 3D view; checkCIF report

## Figures and Tables

**Table 1:** Hydrogen-bond geometry (Å, °) *Cg*1, *Cg*2, *Cg*3, *Cg*4 and *Cg*5 are the centroids of the C16*B*–C18*B*/N1*B*/N2*B*, C1*B*–C6*B*, C16*A*–C18*A*/N1*A*/N2*A*, C1*A*–C6*A* and C10*A*–C15*A* rings, respectively.

*D*—H⋯*A*	*D*—H	H⋯*A*	*D*⋯*A*	*D*—H⋯*A*
C17*B*—H17*B*⋯O1*A*	0.93	2.27	3.163 (3)	160
C20*A*—H20*C*⋯F1*A*	0.96	2.53	3.377 (2)	147
C2*A*—H2*AA*⋯*Cg*5^i^	0.93	2.81	3.727 (2)	167
C13*A*—H13*A*⋯*Cg*4^ii^	0.93	2.98	3.7764 (19)	144
C13*B*—H13*B*⋯*Cg*2^ii^	0.93	2.93	3.716 (2)	143

Symmetry codes: (i) 
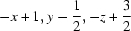
; (ii) 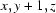.

**Fig. 1.**
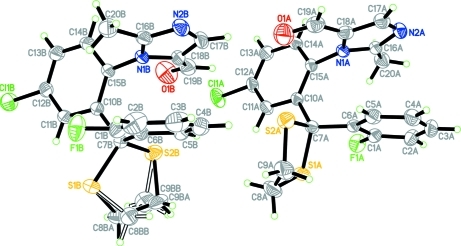
The structure of the aymmetric unit of the title compound, showing 40% probability displacement ellipsoids and the atom-numbering scheme. Open bonds indicate the minor disorder component. Hydrogen atoms are shown as spheres of arbitrary radius.

**Fig. 2.**
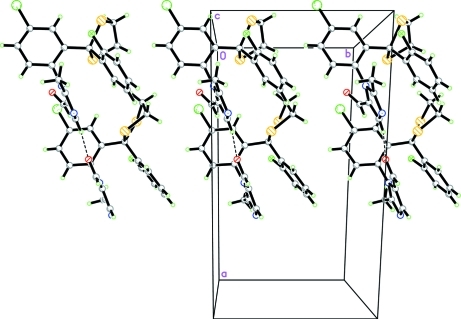
The crystal packing of the major disorder component of the title compound, viewed down the c axis. Weak C—H···O interactions are shown as dashed lines.

## supplementary crystallographic information

### Comment

Imidazole is a constituent part of some very important compounds, such as purine, adenine, xanthine, guanine and co-enzyme A, and many drugs contain the imidazole ring. Imidazole derivatives have occupied a unique place in the field of medicinal chemistry. They have a wide range of biological properties, such as antifungal (Hori *et al.*, 2000; Mamolo *et al.*, 2004) and antibacterial (Khabnadideh *et al.*, 2003) activities. They are well known analgesic (Ucucu *et al.*, 2001), anti-inflammatory (Yesilada *et al.*, 2004), anthelmintic (Dutta *et al.*, 2009), antiparasitic (Quattara *et al.*, 1987), as well as antimicrobial (Sengupta & Bhattacharya, 1983) agents. In view of its interesting biological and pharmacological activities, the title compound was synthesized to evaluate its biological activities; its crystal structure is reported here.

Fig. 1 shows the asymmetric unit, which consists of two molecules *A* and *B* of C_20_H_16_ClFN_2_OS_2_. In molecule *B*, the dithiolan ring is disordered over two sites, the major component *BA* and the minor component *BB* (Fig. 1 ), having a refined site-occupancy ratio of 0.849 (9)/0.151 (10). In molecule *A*, the imidazole ring makes dihedral angles of 79.56 (9) and 18.45 (9)° with the 4-chlorophenyl and 2-fluorophenyl rings, respectively, whereas the corresponding angles in molecule *B* are 82.72 (9) and 17.39 (10)°. The bond lengths are in normal ranges (Allen *et al.*, 1987).

The conformations of the dithiolan ring (C7–C9/S1–S2) in molecules *A* and *B* are different. In molecule *A*, the dithiolan ring is in an envelope conformation with the flap atom, C9A, 0.327 (2) Å out-of-plane, and puckering parameters Q = 0.517 (2) Å and φ = 106.7 (2)° (Cremer & Pople, 1975). In molecule *B*, the dithiolan rings of both major and minor disorder components are in half-chair conformations, with puckering parameters Q = 0.536 (3) Å and φ = 263.5 (3)° for the major component and Q = 0.544 (15) Å and φ = 155 (2)° for the minor component .

In the crystal packing (Fig. 2), the two molecules of the asymmetric unit are linked by a weak C—H···O interaction (Table 1) involving the imidazole and aldehyde groups (C17B—H17B···O1A), and these linked molecules are stacked along the *b* axis by π–π interactions with a *Cg*1···*Cg*3^iii^ distance of 3.4922 (11) Å (symmetry code; 1-x, 1-y, 2-z). In addition, π–π interactions between the imidazole and 2-fluorophenyl rings are also observed, with distances of *Cg*1···*Cg*2 = 3.4867 (11) Å and *Cg*3···*Cg*4 = 3.4326 (10) Å. The crystal structure is consolidated and stabilized by weak C—H···π interactions (Table 1). Cl···S [3.5185 (8) Å], C···O [3.192 (3) Å] and C···C [3.326 (2)–3.393 (3) Å] short contacts are also observed.

### Experimental

To a 500 ml three-necked flask containing isopropyl alcohol (123 ml), 4-chloro-2[2-(2-fluorophenyl)-1,3-dithiolan-2-yl]-*N*-(1-aminoethylidene) benzenamine (20 g, 0.054 mole) was added, then followed by acetic acid and triethylamine (1.1 molar equivalent of each). A solution of bromomalonaldehyde (8.65 g, 0.055 mole) in 100 ml of isopropyl alcohol was added and refluxed for 6 hrs. The mixture was concentrated under vacuum at 308–313 K and the residue was treated with water (200 ml), followed by extraction with dichloromethane (100 ml). The organic layer was concentrated and the product isolated. Yellow block-shaped single crystals of the title compound suitable for *X*-ray structure determination were recrystallized from ethanol by the slow evaporation of the solvent at room temperature over a period of several days, Mp. 431–433 K.

### Refinement

H atoms were positioned geometrically and allowed to ride on their parent atoms, with d(C—H) = 0.93 Å for Csp^2^, 0.97 Å for methylene C and 0.96 Å for methyl C atoms. *U*_iso_(H) = x*U*_eq_(C), where x = 1.5 for methyl H and 1.2 for all other H atoms. A rotating group model was used for the methyl groups.

### Crystal data

**Table:**

C_20_H_16_ClFN_2_OS_2_	*F*(000) = 1728
*M**_r_* = 418.92	*D*_x_ = 1.465 Mg m^−^^3^
Monoclinic, *P*2_1_/*c*	Melting point = 431–433 K
Hall symbol: -P 2ybc	Mo *K*α radiation, λ = 0.71073 Å
*a* = 18.5654 (3) Å	Cell parameters from 11058 reflections
*b* = 9.2730 (1) Å	θ = 2.3–30.0°
*c* = 24.7174 (4) Å	µ = 0.44 mm^−^^1^
β = 116.807 (1)°	*T* = 297 K
*V* = 3797.96 (10) Å^3^	Block, yellow
*Z* = 8	0.57 × 0.52 × 0.43 mm

### Data collection

**Table:**

Bruker SMART APEXII CCD area-detector diffractometer	11058 independent reflections
Radiation source: sealed tube	8238 reflections with *I* > 2σ(*I*)
graphite	*R*_int_ = 0.025
φ and ω scans	θ_max_ = 30.0°, θ_min_ = 2.3°
Absorption correction: multi-scan (*SADABS*; Bruker, 2005)	*h* = −25→26
*T*_min_ = 0.787, *T*_max_ = 0.833	*k* = −11→13
42050 measured reflections	*l* = −34→34

### Refinement

**Table:**

Refinement on *F*^2^	Primary atom site location: structure-invariant direct methods
Least-squares matrix: full	Secondary atom site location: difference Fourier map
*R*[*F*^2^ > 2σ(*F*^2^)] = 0.040	Hydrogen site location: inferred from neighbouring sites
*wR*(*F*^2^) = 0.110	H-atom parameters constrained
*S* = 1.01	*w* = 1/[σ^2^(*F*_o_^2^) + (0.0494*P*)^2^ + 1.2334*P*] where *P* = (*F*_o_^2^ + 2*F*_c_^2^)/3
11058 reflections	(Δ/σ)_max_ = 0.001
498 parameters	Δρ_max_ = 0.47 e Å^−^^3^
0 restraints	Δρ_min_ = −0.29 e Å^−^^3^

### Special details

**Table:**

Geometry. All e.s.d.'s (except the e.s.d. in the dihedral angle between two l.s. planes) are estimated using the full covariance matrix. The cell e.s.d.'s are taken into account individually in the estimation of e.s.d.'s in distances, angles and torsion angles; correlations between e.s.d.'s in cell parameters are only used when they are defined by crystal symmetry. An approximate (isotropic) treatment of cell e.s.d.'s is used for estimating e.s.d.'s involving l.s. planes.
Refinement. Refinement of *F*^2^ against ALL reflections. The weighted *R*-factor *wR* and goodness of fit *S* are based on *F*^2^, conventional *R*-factors *R* are based on *F*, with *F* set to zero for negative *F*^2^. The threshold expression of *F*^2^ > σ(*F*^2^) is used only for calculating *R*-factors(gt) *etc*. and is not relevant to the choice of reflections for refinement. *R*-factors based on *F*^2^ are statistically about twice as large as those based on *F*, and *R*- factors based on ALL data will be even larger.

### Fractional atomic coordinates and isotropic or equivalent isotropic displacement parameters (Å^2^)

**Table:**

	*x*	*y*	*z*	*U*_iso_*/*U*_eq_	Occ. (<1)
Cl1A	0.25942 (3)	0.62718 (5)	0.73463 (3)	0.06226 (14)	
S1A	0.30891 (3)	0.06420 (5)	0.76209 (2)	0.04776 (11)	
S2A	0.35008 (3)	0.12181 (5)	0.89061 (2)	0.04827 (11)	
F1A	0.45561 (7)	0.10894 (12)	0.75343 (5)	0.0543 (3)	
O1A	0.45746 (10)	0.3552 (2)	0.97799 (8)	0.0808 (5)	
N1A	0.54863 (7)	0.30739 (13)	0.90641 (6)	0.0333 (3)	
N2A	0.67475 (8)	0.22391 (16)	0.94763 (7)	0.0462 (3)	
C1A	0.49232 (10)	0.03084 (17)	0.80578 (8)	0.0411 (3)	
C2A	0.55553 (11)	−0.0572 (2)	0.81286 (10)	0.0536 (5)	
H2AA	0.5715	−0.0656	0.7823	0.064*	
C3A	0.59483 (12)	−0.1329 (2)	0.86655 (11)	0.0603 (5)	
H3AA	0.6381	−0.1927	0.8727	0.072*	
C4A	0.56967 (12)	−0.1193 (2)	0.91058 (10)	0.0562 (5)	
H4AA	0.5967	−0.1692	0.9469	0.067*	
C5A	0.50453 (10)	−0.03238 (17)	0.90177 (8)	0.0439 (4)	
H5AA	0.4878	−0.0263	0.9319	0.053*	
C6A	0.46387 (9)	0.04589 (15)	0.84846 (7)	0.0352 (3)	
C7A	0.38948 (9)	0.13927 (16)	0.83468 (7)	0.0342 (3)	
C8A	0.23702 (12)	0.0129 (3)	0.78845 (10)	0.0632 (5)	
H8AA	0.2005	0.0923	0.7835	0.076*	
H8AB	0.2055	−0.0688	0.7654	0.076*	
C9A	0.28267 (13)	−0.0262 (2)	0.85399 (11)	0.0617 (5)	
H9AA	0.3130	−0.1145	0.8587	0.074*	
H9AB	0.2459	−0.0405	0.8716	0.074*	
C10A	0.40297 (9)	0.30204 (16)	0.82977 (7)	0.0328 (3)	
C11A	0.33688 (9)	0.38578 (18)	0.79124 (8)	0.0391 (3)	
H11A	0.2872	0.3414	0.7689	0.047*	
C12A	0.34365 (9)	0.53227 (18)	0.78570 (8)	0.0403 (3)	
C13A	0.41532 (10)	0.60404 (18)	0.81777 (8)	0.0449 (4)	
H13A	0.4193	0.7029	0.8135	0.054*	
C14A	0.48118 (10)	0.52407 (17)	0.85662 (8)	0.0418 (4)	
H14A	0.5303	0.5702	0.8788	0.050*	
C15A	0.47569 (9)	0.37588 (16)	0.86333 (7)	0.0330 (3)	
C16A	0.61461 (9)	0.27685 (17)	0.89769 (7)	0.0368 (3)	
C17A	0.64670 (11)	0.22370 (19)	0.98946 (8)	0.0463 (4)	
H17A	0.6764	0.1917	1.0291	0.056*	
C18A	0.56970 (10)	0.27589 (17)	0.96678 (7)	0.0392 (3)	
C19A	0.52354 (13)	0.3053 (2)	0.99857 (9)	0.0527 (4)	
H19A	0.5476	0.2831	1.0397	0.063*	
C20A	0.61982 (11)	0.3074 (2)	0.84079 (8)	0.0501 (4)	
H20A	0.6601	0.2465	0.8384	0.075*	
H20B	0.6342	0.4066	0.8403	0.075*	
H20C	0.5685	0.2887	0.8068	0.075*	
Cl1B	−0.11743 (3)	0.82366 (6)	0.82366 (2)	0.06218 (14)	
S1B	−0.06051 (3)	0.25797 (5)	0.84138 (2)	0.04791 (11)	
S2B	0.07738 (3)	0.33592 (5)	0.81837 (2)	0.04785 (11)	
F1B	0.00272 (8)	0.29431 (15)	0.97221 (5)	0.0681 (3)	
O1B	0.20278 (10)	0.6108 (2)	0.86047 (7)	0.0772 (5)	
N1B	0.18622 (7)	0.50963 (14)	0.96952 (6)	0.0348 (3)	
N2B	0.28898 (9)	0.41459 (16)	1.04951 (7)	0.0475 (3)	
C1B	0.06791 (12)	0.2203 (2)	0.97587 (8)	0.0479 (4)	
C2B	0.10655 (16)	0.1275 (2)	1.02414 (9)	0.0659 (6)	
H2BA	0.0872	0.1141	1.0525	0.079*	
C3B	0.17401 (15)	0.0555 (2)	1.02951 (10)	0.0688 (6)	
H3BA	0.2010	−0.0071	1.0618	0.083*	
C4B	0.20137 (12)	0.0759 (2)	0.98734 (10)	0.0593 (5)	
H4BA	0.2476	0.0281	0.9913	0.071*	
C5B	0.16075 (10)	0.16754 (18)	0.93844 (8)	0.0448 (4)	
H5BA	0.1797	0.1786	0.9097	0.054*	
C6B	0.09254 (9)	0.24306 (16)	0.93159 (7)	0.0357 (3)	
C7B	0.04282 (9)	0.33975 (17)	0.87717 (7)	0.0336 (3)	
C10B	0.04177 (9)	0.50073 (16)	0.89252 (6)	0.0321 (3)	
C11B	−0.02824 (9)	0.58123 (18)	0.86003 (7)	0.0374 (3)	
H11B	−0.0750	0.5347	0.8329	0.045*	
C12B	−0.02917 (10)	0.72888 (18)	0.86748 (7)	0.0401 (3)	
C13B	0.03757 (11)	0.80291 (19)	0.90805 (8)	0.0464 (4)	
H13B	0.0361	0.9022	0.9128	0.056*	
C14B	0.10674 (10)	0.72464 (18)	0.94136 (8)	0.0445 (4)	
H14B	0.1525	0.7718	0.9696	0.053*	
C15B	0.10945 (9)	0.57683 (17)	0.93361 (7)	0.0342 (3)	
C16B	0.21586 (10)	0.47013 (18)	1.02890 (7)	0.0399 (3)	
C17B	0.30732 (10)	0.42030 (19)	1.00227 (9)	0.0468 (4)	
H17B	0.3557	0.3880	1.0039	0.056*	
C18B	0.24570 (9)	0.47949 (17)	0.95176 (8)	0.0396 (3)	
C19B	0.24897 (12)	0.5274 (2)	0.89772 (9)	0.0532 (4)	
H19B	0.2906	0.4909	0.8906	0.064*	
C20B	0.17221 (12)	0.4943 (3)	1.06578 (9)	0.0579 (5)	
H20D	0.1919	0.4284	1.0993	0.087*	
H20E	0.1155	0.4786	1.0411	0.087*	
H20F	0.1809	0.5915	1.0807	0.087*	
C8BA	−0.0620 (2)	0.1950 (5)	0.77250 (17)	0.0664 (10)	0.849 (10)
H8BA	−0.0914	0.1048	0.7604	0.080*	0.849 (10)
H8BB	−0.0889	0.2650	0.7404	0.080*	0.849 (10)
C9BA	0.0235 (2)	0.1734 (4)	0.78267 (16)	0.0593 (9)	0.849 (10)
H9BA	0.0250	0.1576	0.7444	0.071*	0.849 (10)
H9BB	0.0474	0.0907	0.8086	0.071*	0.849 (10)
C8BB	−0.0388 (12)	0.1405 (19)	0.7960 (9)	0.053 (4)*	0.151 (10)
H8BC	−0.0007	0.0679	0.8208	0.064*	0.151 (10)
H8BD	−0.0877	0.0923	0.7676	0.064*	0.151 (10)
C9BB	−0.0027 (15)	0.228 (3)	0.7614 (11)	0.075 (6)*	0.151 (10)
H9BC	−0.0434	0.2900	0.7314	0.090*	0.151 (10)
H9BD	0.0188	0.1649	0.7410	0.090*	0.151 (10)

### Atomic displacement parameters (Å^2^)

**Table:**

	*U*^11^	*U*^22^	*U*^33^	*U*^12^	*U*^13^	*U*^23^
Cl1A	0.0383 (2)	0.0507 (3)	0.0845 (4)	0.0141 (2)	0.0160 (2)	0.0254 (2)
S1A	0.0388 (2)	0.0450 (2)	0.0457 (2)	−0.00736 (18)	0.00682 (18)	−0.00719 (18)
S2A	0.0500 (3)	0.0469 (2)	0.0538 (3)	−0.0069 (2)	0.0286 (2)	−0.0025 (2)
F1A	0.0612 (7)	0.0563 (6)	0.0475 (6)	0.0087 (5)	0.0264 (5)	0.0081 (5)
O1A	0.0655 (10)	0.1031 (14)	0.0858 (12)	−0.0056 (10)	0.0447 (9)	−0.0174 (10)
N1A	0.0274 (6)	0.0310 (6)	0.0355 (6)	−0.0008 (5)	0.0090 (5)	−0.0007 (5)
N2A	0.0352 (7)	0.0425 (8)	0.0498 (8)	0.0069 (6)	0.0093 (6)	0.0016 (6)
C1A	0.0399 (8)	0.0325 (8)	0.0464 (9)	−0.0002 (7)	0.0155 (7)	−0.0011 (7)
C2A	0.0495 (10)	0.0407 (10)	0.0730 (13)	0.0015 (8)	0.0299 (10)	−0.0093 (9)
C3A	0.0413 (10)	0.0355 (9)	0.0924 (16)	0.0082 (8)	0.0199 (10)	−0.0023 (10)
C4A	0.0462 (10)	0.0342 (9)	0.0649 (12)	0.0039 (8)	0.0046 (9)	0.0097 (8)
C5A	0.0442 (9)	0.0304 (8)	0.0469 (9)	−0.0026 (7)	0.0116 (8)	0.0054 (7)
C6A	0.0329 (7)	0.0236 (7)	0.0421 (8)	−0.0023 (6)	0.0107 (6)	−0.0008 (6)
C7A	0.0303 (7)	0.0304 (7)	0.0369 (7)	−0.0023 (6)	0.0106 (6)	0.0005 (6)
C8A	0.0393 (10)	0.0584 (12)	0.0797 (15)	−0.0132 (9)	0.0161 (10)	−0.0025 (11)
C9A	0.0541 (12)	0.0507 (11)	0.0834 (15)	−0.0127 (9)	0.0336 (11)	0.0067 (10)
C10A	0.0286 (7)	0.0283 (7)	0.0390 (7)	0.0003 (6)	0.0132 (6)	0.0005 (6)
C11A	0.0260 (7)	0.0371 (8)	0.0484 (9)	0.0002 (6)	0.0116 (7)	0.0037 (7)
C12A	0.0310 (8)	0.0368 (8)	0.0495 (9)	0.0078 (6)	0.0148 (7)	0.0082 (7)
C13A	0.0444 (9)	0.0281 (8)	0.0575 (10)	0.0018 (7)	0.0190 (8)	0.0032 (7)
C14A	0.0332 (8)	0.0323 (8)	0.0506 (9)	−0.0043 (6)	0.0106 (7)	−0.0018 (7)
C15A	0.0272 (7)	0.0301 (7)	0.0374 (7)	0.0024 (6)	0.0107 (6)	−0.0002 (6)
C16A	0.0307 (7)	0.0320 (7)	0.0420 (8)	0.0007 (6)	0.0112 (6)	−0.0025 (6)
C17A	0.0457 (9)	0.0394 (9)	0.0389 (8)	0.0040 (7)	0.0060 (7)	0.0040 (7)
C18A	0.0420 (9)	0.0329 (8)	0.0373 (8)	−0.0035 (7)	0.0132 (7)	−0.0004 (6)
C19A	0.0556 (12)	0.0522 (11)	0.0548 (11)	−0.0113 (9)	0.0287 (10)	−0.0083 (9)
C20A	0.0441 (10)	0.0569 (11)	0.0514 (10)	−0.0008 (8)	0.0234 (8)	−0.0001 (8)
Cl1B	0.0466 (3)	0.0566 (3)	0.0570 (3)	0.0220 (2)	0.0001 (2)	0.0008 (2)
S1B	0.0339 (2)	0.0497 (3)	0.0522 (2)	−0.00964 (18)	0.01240 (19)	−0.0126 (2)
S2B	0.0566 (3)	0.0502 (3)	0.0435 (2)	−0.0079 (2)	0.0286 (2)	−0.00982 (19)
F1B	0.0733 (8)	0.0838 (9)	0.0640 (7)	0.0218 (7)	0.0458 (7)	0.0078 (6)
O1B	0.0712 (10)	0.0979 (13)	0.0585 (9)	0.0014 (9)	0.0257 (8)	0.0205 (9)
N1B	0.0249 (6)	0.0350 (7)	0.0365 (6)	0.0012 (5)	0.0069 (5)	−0.0032 (5)
N2B	0.0362 (7)	0.0430 (8)	0.0487 (8)	0.0058 (6)	0.0064 (6)	0.0042 (6)
C1B	0.0539 (11)	0.0463 (10)	0.0429 (9)	0.0055 (8)	0.0213 (8)	0.0000 (8)
C2B	0.0898 (17)	0.0588 (13)	0.0454 (10)	0.0042 (12)	0.0271 (11)	0.0068 (9)
C3B	0.0797 (15)	0.0440 (11)	0.0531 (12)	0.0071 (11)	0.0038 (11)	0.0071 (9)
C4B	0.0476 (11)	0.0357 (9)	0.0721 (13)	0.0082 (8)	0.0072 (10)	−0.0021 (9)
C5B	0.0380 (8)	0.0334 (8)	0.0568 (10)	0.0018 (7)	0.0160 (8)	−0.0068 (7)
C6B	0.0359 (8)	0.0299 (7)	0.0364 (7)	0.0001 (6)	0.0119 (6)	−0.0053 (6)
C7B	0.0295 (7)	0.0355 (8)	0.0321 (7)	−0.0017 (6)	0.0106 (6)	−0.0063 (6)
C10B	0.0295 (7)	0.0340 (8)	0.0296 (7)	0.0009 (6)	0.0107 (6)	−0.0023 (6)
C11B	0.0293 (7)	0.0421 (8)	0.0316 (7)	0.0022 (6)	0.0055 (6)	−0.0038 (6)
C12B	0.0332 (8)	0.0421 (9)	0.0358 (8)	0.0102 (7)	0.0075 (6)	0.0016 (7)
C13B	0.0429 (9)	0.0335 (8)	0.0520 (10)	0.0047 (7)	0.0120 (8)	−0.0036 (7)
C14B	0.0343 (8)	0.0374 (8)	0.0490 (9)	−0.0004 (7)	0.0074 (7)	−0.0078 (7)
C15B	0.0262 (7)	0.0358 (8)	0.0340 (7)	0.0023 (6)	0.0077 (6)	−0.0023 (6)
C16B	0.0342 (8)	0.0367 (8)	0.0378 (8)	−0.0001 (6)	0.0065 (7)	−0.0031 (6)
C17B	0.0311 (8)	0.0385 (9)	0.0629 (11)	0.0052 (7)	0.0142 (8)	0.0012 (8)
C18B	0.0309 (7)	0.0342 (8)	0.0508 (9)	−0.0008 (6)	0.0159 (7)	−0.0034 (7)
C19B	0.0470 (10)	0.0601 (12)	0.0551 (11)	−0.0074 (9)	0.0253 (9)	−0.0050 (9)
C20B	0.0550 (11)	0.0702 (13)	0.0440 (10)	0.0043 (10)	0.0184 (9)	−0.0057 (9)
C8BA	0.0594 (17)	0.074 (2)	0.0480 (16)	−0.0147 (15)	0.0090 (14)	−0.0239 (16)
C9BA	0.0722 (19)	0.0551 (17)	0.0508 (16)	−0.0098 (14)	0.0279 (15)	−0.0260 (13)

### Geometric parameters (Å, °)

**Table:**

Cl1A—C12A	1.7415 (16)	S2B—C9BA	1.804 (3)
S1A—C8A	1.794 (2)	S2B—C9BB	1.82 (2)
S1A—C7A	1.8764 (16)	S2B—C7B	1.8349 (15)
S2A—C9A	1.802 (2)	F1B—C1B	1.358 (2)
S2A—C7A	1.8396 (16)	O1B—C19B	1.212 (3)
F1A—C1A	1.367 (2)	N1B—C16B	1.365 (2)
O1A—C19A	1.190 (2)	N1B—C18B	1.388 (2)
N1A—C16A	1.3659 (19)	N1B—C15B	1.4375 (19)
N1A—C18A	1.392 (2)	N2B—C16B	1.321 (2)
N1A—C15A	1.4389 (19)	N2B—C17B	1.357 (2)
N2A—C16A	1.330 (2)	C1B—C6B	1.379 (2)
N2A—C17A	1.351 (2)	C1B—C2B	1.381 (3)
C1A—C2A	1.374 (2)	C2B—C3B	1.372 (3)
C1A—C6A	1.383 (2)	C2B—H2BA	0.9300
C2A—C3A	1.383 (3)	C3B—C4B	1.363 (3)
C2A—H2AA	0.9300	C3B—H3BA	0.9300
C3A—C4A	1.370 (3)	C4B—C5B	1.389 (3)
C3A—H3AA	0.9300	C4B—H4BA	0.9300
C4A—C5A	1.387 (3)	C5B—C6B	1.389 (2)
C4A—H4AA	0.9300	C5B—H5BA	0.9300
C5A—C6A	1.391 (2)	C6B—C7B	1.531 (2)
C5A—H5AA	0.9300	C7B—C10B	1.542 (2)
C6A—C7A	1.533 (2)	C10B—C11B	1.397 (2)
C7A—C10A	1.544 (2)	C10B—C15B	1.399 (2)
C8A—C9A	1.495 (3)	C11B—C12B	1.383 (2)
C8A—H8AA	0.9700	C11B—H11B	0.9300
C8A—H8AB	0.9700	C12B—C13B	1.375 (2)
C9A—H9AA	0.9700	C13B—C14B	1.378 (2)
C9A—H9AB	0.9700	C13B—H13B	0.9300
C10A—C11A	1.400 (2)	C14B—C15B	1.388 (2)
C10A—C15A	1.403 (2)	C14B—H14B	0.9300
C11A—C12A	1.377 (2)	C16B—C20B	1.485 (2)
C11A—H11A	0.9300	C17B—C18B	1.371 (2)
C12A—C13A	1.375 (2)	C17B—H17B	0.9300
C13A—C14A	1.381 (2)	C18B—C19B	1.435 (3)
C13A—H13A	0.9300	C19B—H19B	0.9300
C14A—C15A	1.393 (2)	C20B—H20D	0.9600
C14A—H14A	0.9300	C20B—H20E	0.9600
C16A—C20A	1.480 (2)	C20B—H20F	0.9600
C17A—C18A	1.367 (2)	C8BA—C9BA	1.504 (5)
C17A—H17A	0.9300	C8BA—H8BA	0.9700
C18A—C19A	1.426 (2)	C8BA—H8BB	0.9700
C19A—H19A	0.9300	C9BA—H9BA	0.9700
C20A—H20A	0.9600	C9BA—H9BB	0.9700
C20A—H20B	0.9600	C8BB—C9BB	1.54 (3)
C20A—H20C	0.9600	C8BB—H8BC	0.9700
Cl1B—C12B	1.7407 (16)	C8BB—H8BD	0.9700
S1B—C8BB	1.737 (15)	C9BB—H9BC	0.9700
S1B—C8BA	1.788 (3)	C9BB—H9BD	0.9700
S1B—C7B	1.8728 (15)		
			
C8A—S1A—C7A	98.85 (9)	C16B—N2B—C17B	105.43 (14)
C9A—S2A—C7A	96.37 (9)	F1B—C1B—C6B	118.32 (15)
C16A—N1A—C18A	106.68 (13)	F1B—C1B—C2B	118.18 (18)
C16A—N1A—C15A	125.89 (13)	C6B—C1B—C2B	123.49 (18)
C18A—N1A—C15A	126.99 (13)	C3B—C2B—C1B	118.9 (2)
C16A—N2A—C17A	105.00 (14)	C3B—C2B—H2BA	120.6
F1A—C1A—C2A	117.88 (16)	C1B—C2B—H2BA	120.6
F1A—C1A—C6A	118.02 (14)	C4B—C3B—C2B	119.8 (2)
C2A—C1A—C6A	124.10 (17)	C4B—C3B—H3BA	120.1
C1A—C2A—C3A	118.32 (19)	C2B—C3B—H3BA	120.1
C1A—C2A—H2AA	120.8	C3B—C4B—C5B	120.48 (19)
C3A—C2A—H2AA	120.8	C3B—C4B—H4BA	119.8
C4A—C3A—C2A	119.60 (17)	C5B—C4B—H4BA	119.8
C4A—C3A—H3AA	120.2	C6B—C5B—C4B	121.45 (19)
C2A—C3A—H3AA	120.2	C6B—C5B—H5BA	119.3
C3A—C4A—C5A	120.96 (18)	C4B—C5B—H5BA	119.3
C3A—C4A—H4AA	119.5	C1B—C6B—C5B	115.88 (16)
C5A—C4A—H4AA	119.5	C1B—C6B—C7B	120.13 (14)
C4A—C5A—C6A	120.95 (18)	C5B—C6B—C7B	123.89 (15)
C4A—C5A—H5AA	119.5	C6B—C7B—C10B	114.84 (12)
C6A—C5A—H5AA	119.5	C6B—C7B—S2B	113.37 (10)
C1A—C6A—C5A	116.03 (15)	C10B—C7B—S2B	105.14 (10)
C1A—C6A—C7A	120.22 (14)	C6B—C7B—S1B	105.13 (10)
C5A—C6A—C7A	123.69 (15)	C10B—C7B—S1B	112.60 (10)
C6A—C7A—C10A	114.07 (12)	S2B—C7B—S1B	105.52 (7)
C6A—C7A—S2A	114.03 (11)	C11B—C10B—C15B	116.49 (14)
C10A—C7A—S2A	106.19 (10)	C11B—C10B—C7B	119.18 (13)
C6A—C7A—S1A	104.64 (10)	C15B—C10B—C7B	124.05 (13)
C10A—C7A—S1A	112.03 (10)	C12B—C11B—C10B	121.13 (15)
S2A—C7A—S1A	105.68 (7)	C12B—C11B—H11B	119.4
C9A—C8A—S1A	107.87 (13)	C10B—C11B—H11B	119.4
C9A—C8A—H8AA	110.1	C13B—C12B—C11B	122.02 (15)
S1A—C8A—H8AA	110.1	C13B—C12B—Cl1B	119.17 (13)
C9A—C8A—H8AB	110.1	C11B—C12B—Cl1B	118.81 (13)
S1A—C8A—H8AB	110.1	C12B—C13B—C14B	117.58 (16)
H8AA—C8A—H8AB	108.4	C12B—C13B—H13B	121.2
C8A—C9A—S2A	106.58 (14)	C14B—C13B—H13B	121.2
C8A—C9A—H9AA	110.4	C13B—C14B—C15B	121.29 (15)
S2A—C9A—H9AA	110.4	C13B—C14B—H14B	119.4
C8A—C9A—H9AB	110.4	C15B—C14B—H14B	119.4
S2A—C9A—H9AB	110.4	C14B—C15B—C10B	121.45 (14)
H9AA—C9A—H9AB	108.6	C14B—C15B—N1B	115.49 (14)
C11A—C10A—C15A	116.47 (14)	C10B—C15B—N1B	123.05 (14)
C11A—C10A—C7A	118.17 (13)	N2B—C16B—N1B	111.43 (15)
C15A—C10A—C7A	125.28 (13)	N2B—C16B—C20B	124.95 (16)
C12A—C11A—C10A	121.56 (15)	N1B—C16B—C20B	123.55 (15)
C12A—C11A—H11A	119.2	N2B—C17B—C18B	111.66 (15)
C10A—C11A—H11A	119.2	N2B—C17B—H17B	124.2
C13A—C12A—C11A	121.89 (15)	C18B—C17B—H17B	124.2
C13A—C12A—Cl1A	119.50 (13)	C17B—C18B—N1B	104.40 (15)
C11A—C12A—Cl1A	118.58 (13)	C17B—C18B—C19B	127.11 (16)
C12A—C13A—C14A	117.65 (15)	N1B—C18B—C19B	127.28 (16)
C12A—C13A—H13A	121.2	O1B—C19B—C18B	126.31 (19)
C14A—C13A—H13A	121.2	O1B—C19B—H19B	116.8
C13A—C14A—C15A	121.52 (15)	C18B—C19B—H19B	116.8
C13A—C14A—H14A	119.2	C16B—C20B—H20D	109.5
C15A—C14A—H14A	119.2	C16B—C20B—H20E	109.5
C14A—C15A—C10A	120.89 (14)	H20D—C20B—H20E	109.5
C14A—C15A—N1A	115.37 (13)	C16B—C20B—H20F	109.5
C10A—C15A—N1A	123.74 (13)	H20D—C20B—H20F	109.5
N2A—C16A—N1A	111.56 (14)	H20E—C20B—H20F	109.5
N2A—C16A—C20A	124.43 (15)	C9BA—C8BA—S1B	108.8 (2)
N1A—C16A—C20A	123.92 (14)	C9BA—C8BA—H8BA	109.9
N2A—C17A—C18A	112.22 (15)	S1B—C8BA—H8BA	109.9
N2A—C17A—H17A	123.9	C9BA—C8BA—H8BB	109.9
C18A—C17A—H17A	123.9	S1B—C8BA—H8BB	109.9
C17A—C18A—N1A	104.49 (14)	H8BA—C8BA—H8BB	108.3
C17A—C18A—C19A	128.22 (17)	C8BA—C9BA—S2B	106.3 (2)
N1A—C18A—C19A	127.03 (16)	C8BA—C9BA—H9BA	110.5
O1A—C19A—C18A	126.8 (2)	S2B—C9BA—H9BA	110.5
O1A—C19A—H19A	116.6	C8BA—C9BA—H9BB	110.5
C18A—C19A—H19A	116.6	S2B—C9BA—H9BB	110.5
C16A—C20A—H20A	109.5	H9BA—C9BA—H9BB	108.7
C16A—C20A—H20B	109.5	C9BB—C8BB—S1B	108.3 (14)
H20A—C20A—H20B	109.5	C9BB—C8BB—H8BC	110.0
C16A—C20A—H20C	109.5	S1B—C8BB—H8BC	110.0
H20A—C20A—H20C	109.5	C9BB—C8BB—H8BD	110.0
H20B—C20A—H20C	109.5	S1B—C8BB—H8BD	110.0
C8BB—S1B—C7B	94.2 (6)	H8BC—C8BB—H8BD	108.4
C8BA—S1B—C7B	98.91 (10)	C8BB—C9BB—S2B	105.7 (16)
C9BA—S2B—C7B	95.08 (10)	C8BB—C9BB—H9BC	110.6
C9BB—S2B—C7B	99.7 (6)	S2B—C9BB—H9BC	110.6
C16B—N1B—C18B	107.07 (13)	C8BB—C9BB—H9BD	110.6
C16B—N1B—C15B	125.95 (13)	S2B—C9BB—H9BD	110.6
C18B—N1B—C15B	126.91 (13)	H9BC—C9BB—H9BD	108.7
			
F1A—C1A—C2A—C3A	177.86 (16)	F1B—C1B—C6B—C7B	−5.0 (2)
C6A—C1A—C2A—C3A	−1.8 (3)	C2B—C1B—C6B—C7B	175.70 (18)
C1A—C2A—C3A—C4A	0.5 (3)	C4B—C5B—C6B—C1B	−0.4 (2)
C2A—C3A—C4A—C5A	1.0 (3)	C4B—C5B—C6B—C7B	−176.88 (16)
C3A—C4A—C5A—C6A	−1.3 (3)	C1B—C6B—C7B—C10B	67.24 (19)
F1A—C1A—C6A—C5A	−178.19 (14)	C5B—C6B—C7B—C10B	−116.45 (16)
C2A—C1A—C6A—C5A	1.4 (2)	C1B—C6B—C7B—S2B	−171.85 (13)
F1A—C1A—C6A—C7A	4.3 (2)	C5B—C6B—C7B—S2B	4.5 (2)
C2A—C1A—C6A—C7A	−176.03 (16)	C1B—C6B—C7B—S1B	−57.09 (17)
C4A—C5A—C6A—C1A	0.1 (2)	C5B—C6B—C7B—S1B	119.22 (15)
C4A—C5A—C6A—C7A	177.49 (15)	C9BA—S2B—C7B—C6B	82.91 (18)
C1A—C6A—C7A—C10A	−67.81 (18)	C9BB—S2B—C7B—C6B	106.1 (10)
C5A—C6A—C7A—C10A	114.93 (16)	C9BA—S2B—C7B—C10B	−150.86 (17)
C1A—C6A—C7A—S2A	169.95 (12)	C9BB—S2B—C7B—C10B	−127.7 (10)
C5A—C6A—C7A—S2A	−7.31 (19)	C9BA—S2B—C7B—S1B	−31.63 (16)
C1A—C6A—C7A—S1A	54.97 (16)	C9BB—S2B—C7B—S1B	−8.4 (10)
C5A—C6A—C7A—S1A	−122.29 (14)	C8BB—S1B—C7B—C6B	−87.5 (7)
C9A—S2A—C7A—C6A	−89.25 (13)	C8BA—S1B—C7B—C6B	−111.47 (19)
C9A—S2A—C7A—C10A	144.28 (12)	C8BB—S1B—C7B—C10B	146.8 (7)
C9A—S2A—C7A—S1A	25.12 (10)	C8BA—S1B—C7B—C10B	122.8 (2)
C8A—S1A—C7A—C6A	119.73 (12)	C8BB—S1B—C7B—S2B	32.6 (7)
C8A—S1A—C7A—C10A	−116.18 (12)	C8BA—S1B—C7B—S2B	8.64 (19)
C8A—S1A—C7A—S2A	−0.96 (11)	C6B—C7B—C10B—C11B	−145.03 (14)
C7A—S1A—C8A—C9A	−30.39 (17)	S2B—C7B—C10B—C11B	89.65 (14)
S1A—C8A—C9A—S2A	51.40 (18)	S1B—C7B—C10B—C11B	−24.74 (17)
C7A—S2A—C9A—C8A	−47.47 (16)	C6B—C7B—C10B—C15B	41.2 (2)
C6A—C7A—C10A—C11A	150.01 (14)	S2B—C7B—C10B—C15B	−84.13 (15)
S2A—C7A—C10A—C11A	−83.54 (15)	S1B—C7B—C10B—C15B	161.48 (12)
S1A—C7A—C10A—C11A	31.36 (17)	C15B—C10B—C11B—C12B	1.5 (2)
C6A—C7A—C10A—C15A	−33.5 (2)	C7B—C10B—C11B—C12B	−172.71 (14)
S2A—C7A—C10A—C15A	92.96 (15)	C10B—C11B—C12B—C13B	−1.7 (3)
S1A—C7A—C10A—C15A	−152.14 (13)	C10B—C11B—C12B—Cl1B	177.78 (12)
C15A—C10A—C11A—C12A	1.4 (2)	C11B—C12B—C13B—C14B	0.4 (3)
C7A—C10A—C11A—C12A	178.23 (15)	Cl1B—C12B—C13B—C14B	−179.10 (14)
C10A—C11A—C12A—C13A	−0.3 (3)	C12B—C13B—C14B—C15B	1.0 (3)
C10A—C11A—C12A—Cl1A	177.61 (13)	C13B—C14B—C15B—C10B	−1.2 (3)
C11A—C12A—C13A—C14A	−0.5 (3)	C13B—C14B—C15B—N1B	177.64 (16)
Cl1A—C12A—C13A—C14A	−178.33 (14)	C11B—C10B—C15B—C14B	−0.1 (2)
C12A—C13A—C14A—C15A	0.0 (3)	C7B—C10B—C15B—C14B	173.82 (15)
C13A—C14A—C15A—C10A	1.2 (3)	C11B—C10B—C15B—N1B	−178.85 (13)
C13A—C14A—C15A—N1A	−178.58 (15)	C7B—C10B—C15B—N1B	−4.9 (2)
C11A—C10A—C15A—C14A	−1.9 (2)	C16B—N1B—C15B—C14B	81.0 (2)
C7A—C10A—C15A—C14A	−178.42 (15)	C18B—N1B—C15B—C14B	−95.36 (19)
C11A—C10A—C15A—N1A	177.91 (14)	C16B—N1B—C15B—C10B	−100.17 (19)
C7A—C10A—C15A—N1A	1.4 (2)	C18B—N1B—C15B—C10B	83.4 (2)
C16A—N1A—C15A—C14A	−75.4 (2)	C17B—N2B—C16B—N1B	0.81 (19)
C18A—N1A—C15A—C14A	96.07 (19)	C17B—N2B—C16B—C20B	−176.26 (18)
C16A—N1A—C15A—C10A	104.81 (18)	C18B—N1B—C16B—N2B	−1.30 (19)
C18A—N1A—C15A—C10A	−83.7 (2)	C15B—N1B—C16B—N2B	−178.28 (14)
C17A—N2A—C16A—N1A	−1.26 (19)	C18B—N1B—C16B—C20B	175.82 (17)
C17A—N2A—C16A—C20A	175.30 (17)	C15B—N1B—C16B—C20B	−1.2 (3)
C18A—N1A—C16A—N2A	2.08 (18)	C16B—N2B—C17B—C18B	0.0 (2)
C15A—N1A—C16A—N2A	174.99 (14)	N2B—C17B—C18B—N1B	−0.8 (2)
C18A—N1A—C16A—C20A	−174.49 (16)	N2B—C17B—C18B—C19B	167.32 (17)
C15A—N1A—C16A—C20A	−1.6 (2)	C16B—N1B—C18B—C17B	1.20 (17)
C16A—N2A—C17A—C18A	−0.1 (2)	C15B—N1B—C18B—C17B	178.14 (15)
N2A—C17A—C18A—N1A	1.30 (19)	C16B—N1B—C18B—C19B	−166.85 (17)
N2A—C17A—C18A—C19A	−173.19 (17)	C15B—N1B—C18B—C19B	10.1 (3)
C16A—N1A—C18A—C17A	−1.97 (17)	C17B—C18B—C19B—O1B	−159.9 (2)
C15A—N1A—C18A—C17A	−174.77 (14)	N1B—C18B—C19B—O1B	5.5 (3)
C16A—N1A—C18A—C19A	172.60 (17)	C8BB—S1B—C8BA—C9BA	−56.2 (13)
C15A—N1A—C18A—C19A	−0.2 (3)	C7B—S1B—C8BA—C9BA	24.1 (4)
C17A—C18A—C19A—O1A	177.3 (2)	S1B—C8BA—C9BA—S2B	−49.0 (4)
N1A—C18A—C19A—O1A	4.0 (3)	C9BB—S2B—C9BA—C8BA	−52.7 (17)
F1B—C1B—C2B—C3B	−177.98 (19)	C7B—S2B—C9BA—C8BA	50.0 (3)
C6B—C1B—C2B—C3B	1.3 (3)	C8BA—S1B—C8BB—C9BB	50.7 (17)
C1B—C2B—C3B—C4B	−0.3 (3)	C7B—S1B—C8BB—C9BB	−51.8 (19)
C2B—C3B—C4B—C5B	−1.0 (3)	S1B—C8BB—C9BB—S2B	50 (2)
C3B—C4B—C5B—C6B	1.4 (3)	C9BA—S2B—C9BB—C8BB	57.1 (19)
F1B—C1B—C6B—C5B	178.35 (16)	C7B—S2B—C9BB—C8BB	−23.2 (19)
C2B—C1B—C6B—C5B	−0.9 (3)		

### Hydrogen-bond geometry (Å, °)

**Table:**

*Cg*1, *Cg*2, *Cg*3, *Cg*4 and *Cg*5 are the centroids of the C16B–C18B/N1B/N2B, C1B–C6B, C16A–C18A/N1A/N2A, C1A–C6A and C10A–C15A rings, respectively.

**Table:**

*D*—H···*A*	*D*—H	H···*A*	*D*···*A*	*D*—H···*A*
C17B—H17B···O1A	0.93	2.27	3.163 (3)	160
C20A—H20C···F1A	0.96	2.53	3.377 (2)	147
C2A—H2AA···Cg5^i^	0.93	2.81	3.727 (2)	167
C13A—H13A···Cg4^ii^	0.93	2.98	3.7764 (19)	144
C13B—H13B···Cg2^ii^	0.93	2.93	3.716 (2)	143

Symmetry codes:  (i) −*x*+1, *y*−1/2, −*z*+3/2; (ii) *x*, *y*+1, *z*.
